# The effect of acclimation to the heat on the resting sweat response

**DOI:** 10.1186/2046-7648-4-S1-A118

**Published:** 2015-09-14

**Authors:** Simon K Delves, John S Kelly, Matthew Gledhill, Rebecca Warke, Joanne L Fallowfield

**Affiliations:** 1 Environmental Medicine & Science, Institute of Naval Medicine Gosport, UK; 2 Department of Adventure Education, University of Chichester, Chichester, UK

## Introduction

Living and working in hot dry environments increases the thermal load on the body with adverse consequences ranging from impaired physical and mental performance to serious heat illness [1]. Acclimatisation can reduce the impact of these consequences, but risks to performance and health can never be completely eliminated. Acclimatisation to a hot environment results in an earlier onset of sweating [2,3], an increased sweat rate for an absolute core temperature [3], and a reduced sweat electrolyte concentration [3], Evaluation of the sweating response is *traditionally *achieved through an exercise-based standardised heat tolerance test undertaken in controlled environmental conditions. The collection of a sweat sample requires a reliable and valid method that avoids contamination of the sample during collection. However, analysis of sweat samples can be time consuming, involving laboratory-based analysis techniques, and not conducive to field use. This study evaluated the sensitivity of a resting sweat collection approach for assessing heat acclimation status in young adults.

## Methods

Eight male volunteers undertook a 6-day laboratory-based heat acclimation protocol (wet-bulb globe temperature index 27.5 (0.5) °C). A standardised Heat Tolerance Test (HTT) was performed the day prior to the acclimation period (HTT_1_), and repeated the day after the acclimation period (HTT_2_). During each HTT, heart rate (HR), rectal temperature (Tr) and skin temperature (Ts), were measured at 5 min intervals. A resting sweat sample was stimulated by pilocarpine iontophoresis and collected by the ELITech, Wescor Macroduct resting sweat collection system prior to each HTT. For the acclimation exercise, each volunteer was instrumented with a Polar heart rate monitor and a rectal thermistor. The acclimation protocol maintained a constant thermal strain model of 90 min of intermittent exercise and rest, where Tr was elevated to 38.8 °C (by undertaking stepping exercise at a work rate equivalent to 50%VO_2_max). On attaining 38.8 °C, the volunteer rested until Tr had decreased to 38.5 °C, after which stepping exercise was recommenced. Differences in variables between HTT_1 _and HTT_2 _were assessed using paired Student *t*-tests. Time series data were analysed with a repeated measures analysis of variance (ANOVA) with post hoc Bonferroni corrected *t*-tests where appropriate.

## Results

Physiological acclimation after the 6-day acclimation period was evident in all volunteers. Heart rate, Tr and Ts responses were lower during HTT_2 _compared with HTT_1 _(P < 0.05), and total exercise time increased between day-1 and day-4, and between day-5 and day-6 (P < 0.05). Resting sweat sodium chloride concentration decreased (58 [14] vs. 46 [16] mmol.L^-1^) between HTT_1 _and HTT_2 _(P < 0.05).

## Discussion

The ability to identify acclimatisation status is desirable when preparing for physical work in the heat. The present study was an exploratory study to evaluate a resting sweat collection approach to assess heat acclimation status. Findings from this study indicate that the resting sweat collection approach may be a good indicator of acclimation status, where changes in sweat sodium chloride concentration were identified between pre and post acclimation.

## Conclusion

The change in resting sweat concentration was consistent with acclimation. Further development is required for this method to be used as an indicator of heat acclimation status.
